# Increased Incidence of Loco-Regional Recurrences Among African American Women with Terminal Stage Breast Cancer

**DOI:** 10.4137/cmo.s988

**Published:** 2008-11-03

**Authors:** Gerardo Colón-Otero, Sherry King, Vandelyn Smith, Carolyn Bieber, Julia Crook, Lawrence A. Solberg, Robert Shannon, Edith A. Perez

**Affiliations:** 1 Mayo Clinic Cancer Center, Cancer Health Disparities Program, Jacksonville, Florida; 2 Community Hospice Northeast (CHNE), Jacksonville, Florida

**Keywords:** oncology, LRR, loco-regional recurrences, breast cancer, African American women, terminal breast cancer, terminal stage breast cancer

## Abstract

A prospective analysis of women with terminal breast cancer admitted to CHNE from November 2006–August 2007 evaluated anecdotal observations that African American (AA) women are likelier than Caucasian women to evidence loco-regional recurrences (LRR). Women with terminal breast cancer who were admitted to CHNE, a not-for-profit hospice serving over 90% of Northeast Florida hospice patients, were eligible for participation. 134 terminal breast cancer patients were assessed by hospice nurses for LRR presence via chest wall examination. 80% of them (107) were Caucasian, 17% (23) were AA and 3% (4) were of other ethnicities. Evidence of LRR were noted in 13% of the women (17/134). The proportion of patients with LRR was higher in AA women than Caucasian women (26% vs. 10%, 6/23 vs. 11/107, respectively), although this difference was not statistically significant (p = 0.08). The majority of Caucasian women with LRR consented to a medical record review, but a minority of AA women consented (8/11 vs. 2/6, respectively, p = 0.16).

**Conclusion:**

Evaluating disparities in breast cancer care outcomes is possible by reviewing data from patients served by hospice programs that aid a majority of patients within a community. This pilot data suggests that AA women with breast cancer have a higher incidence of loco-regional failure as a component of their terminal breast cancer disease than Caucasian women. A smaller proportion of AA patients and families agreed to participate in a medical record review study than Caucasians. Larger studies are necessary to confirm these findings, to elucidate factors contributing to disparities and to develop potential solutions.

## Introduction

CHNE is a not-for-profit hospice that serves over 90% of hospice patients in Northeast Florida, which includes Duval, Nassau, St. Johns, and Putnam counties. The enrollment of a majority of hospice patients in a given community by a single, not-for profit program facilitates the conduct of community-based studies of terminal cancer patients that may show evidence of disparities in cancer care. Unfortunately, disparities research involving hospice patients has not been frequently reported. A Pub Med search conducted October, 2008, of “hospice and disparities” yielded only 26 articles published since 1989 in the English-language literature.

Anecdotal observations of terminal breast cancer patients cared for at CHNE suggested that, upon enrollment to hospice, African American (AA) patients were more likely to have evidence of loco-regional recurrence (LRR) as part of the extent of their disease than Caucasian patients. Multiple factors have been associated with a higher incidence of LRR among breast cancer patients including younger age, tumor size, tumor grade, hormone receptor status, her-2 status, lymph node involvement, presence of positive surgical margins and presence of lymphovascular space invasion.^1^–^4^ Compared to Caucasian women with breast cancer, AA women are less likely to receive adjuvant treatments for their breast cancer, which puts them at a higher risk for LRR.^5^ In part, these disparities are secondary to a higher incidence of co-morbidities and a higher incidence of lack of insurance in AA women.^5^ Pierce et al. reported that AAs had a higher incidence of regional failure as part of the first site of failure than Caucasians (16% vs. 4%).^1^ Fowble et al. reported that women diagnosed at ages younger than 35 years old had a 24% risk of LRR; this was significantly higher than that of older women.^2^ AA women are more likely to develop breast cancer at a younger age than Caucasian women.^6^ Triple negative (estrogen receptor, progesterone receptor, and her-2-neu negative) breast cancers typically have a more aggressive behavior with poorer prognosis than other breast cancers and are more common in younger AA women than older AA women.^3^

We sought to provide further evidence that supports anecdotal observations of a higher incidence of loco-regional failure as part of the terminal disease manifestations of AA patients entering CHNE hospice programs. If data supporting these observations was obtained, we planned to follow up this prospective pilot study with a more detailed analysis involving a larger cohort of patients.

## Methods

A Mayo Clinic, IRB-approved prospective study of breast cancer patients enrolled in CHNE was performed from December 2006 to August 2007. The study investigated LRR incidence as part of terminal-stage breast cancer manifestation to determine if it was higher in AA or Caucasian patients. A patient was classified as having terminal-stage breast cancer if the patient had metastatic breast cancer and was enrolled in hospice and did not receive additional chemotherapy or radiation treatments. Hospice nurses evaluated the patients for the presence of LRR upon enrollment to CHNE by performing a physical examination of the chest wall. Nurses also asked patients or the patients’ relatives for consent to obtain medical records for review and for patients or their relatives to meet with a Mayo Clinic research coordinator for an interview. An in-depth review of the medical records of 10 consenting patients with LRR was performed. This pilot study was designed with the intent of accruing 10 patients who would consent to a medical record review; sample size was not based on any power calculations. Fisher’s exact test was used to compare proportions of patients with LRR between groups as well as proportions consenting to a medical record review.

## Results

A total of 134 consecutive women with terminal stage breast cancer who were enrolled in CHNE from December 2006 to August 2007 were evaluated. 80% (107 patients) were Caucasian, 17% (23 patients) were AA, and 3% (4 patients) were of other ethnicities. This distribution of ethnicities is similar to that of the general population of Northeast Florida, though the percentage of AAs in Northeast Florida is somewhat higher and estimated at 27%. Evidence of LRR was noted in 13% of the women (17/134 patients). The incidence of LRR was higher in AA women than Caucasian women (26% vs. 10%, 6/23 vs. 11/107, respectively, p = 0.08). The majority of Caucasian women with LRR consented to a review of their medical records, but a minority of the AA women consented (8/11 vs. 2/6 patients respectively, p = 0.16).

We undertook an extensive medical record review of the 2 AA and 8 Caucasian patients who consented and interviewed patients or their immediate relatives to ascertain which of the described risk factors for LRR were present. Delays in initial diagnosis due to lack of insurance, patient’s denial of breast cancer diagnosis, the presence of locally advanced breast cancer at diagnosis, and late relapses (>5 years) of breast cancer were noted as potentially contributing factors in these 10 cases (Table 1).

## Discussion

This exploratory pilot study suggests that AA patients with terminal breast cancer may be more likely than Caucasian patients to have evidence of LRR, which supports the impression of anecdotal observations. A medical record review of 10 patients with LRR suggests that common, potential factors contributing to LRR in the patient population we reviewed are delays in diagnosis due to lack of insurance and fear of breast cancer diagnosis. Locally advanced breast cancer was present at initial diagnosis in 3 of the cases and this likely contributed to LRR development. Interestingly, 4 patients experienced late (>5 years) LRR; these results stress the importance of ongoing surveillance in the care of breast cancer survivors. Many cancer survivors consider their cancer cured if no relapse occurs in five years. It is conceivable that both patients and their care providers were not as vigilant of LRR after 5 years. Three of the patients with LRR whose medical records were reviewed were diagnosed more than 10 years after the original diagnosis of breast cancer.

The availability of a not-for profit, community-based hospice program that serves a majority of patients from one region makes it feasible to analyze these patients and determine the possible presence of disparities in outcome as demonstrated by our study. A partnership between cancer centers and hospice programs can lead to research studies that will eliminate disparities and improve the palliative care of terminal-stage cancer patients.

The results also suggest that AA families may be less willing to consent to study participation than Caucasian families. Obtaining consents for research studies during the terminal stages of cancer is difficult given how stressful this clinical situation is for patients and their families. Patients may be sedated and unable to provide consent and families may not be certain about the wishes of patients or be comfortable acting as surrogates for consent. The number of patients evaluated in our study was too small to reach any definite conclusions and further studies are warranted to follow-up on our findings.

There are several limitations to this study. Sample sizes were small and no power calculations were part of the original design of this pilot study. The study was conceived as a hypothesis-generating pilot study with the understanding that a larger, prospective study will be needed to confirm the significance of the pilot observations. Such a study is currently underway. Even though CHNE provides hospice care to 90% of the Northeast Florida population it is possible that different qualitative results would result from a study including patients served by other hospice providers. Characteristics of women with terminal breast cancer who are not enrolled in hospice may also differ from those in hospice, and differential proportions of AA and Caucasian women may be enrolled in hospice. These possibilities limit our ability to generalize these findings to the breast cancer population of Northeast Florida. In addition, patients with LRR were examined and results were documented by nurses; confirmation by radiographic studies or medical examinations were not undertaken, thus the validity of the data may be limited.

Significant morbidity can be associated with LRR. Several patients developed painful ulcerated lesions and associated infections. It is important that palliative management of patients with metastatic disease include efforts to minimize the risk of LRR even in the presence of disseminated disease. This is particularly important given the increasing median survival of patients with metastatic breast cancer.

## Conclusions

Evaluation of disparities in outcomes of breast cancer care in a given region is facilitated with the existence of a hospice program that serves the majority of the patients within that community. AA women with breast cancer seem to have a higher incidence of loco-regional failure as a component of their terminal breast cancer disease than Caucasian women. A smaller proportion of AA patients and families agreed to participate in a medical record review study than Caucasian patients and families.

Multiple factors likely contribute to LRR in the patients studied, including lack of insurance, which delays diagnosis, and a lack of awareness about the benefits of timely breast cancer management. Programs that improve the education of this population about the importance of early diagnosis and programs that improve access to care of the general population are likely to ameliorate the problems. Further studies with a larger cohort of patients are indicated in order to confirm these findings as well as to hopefully elucidate the main factors contributing to disparities and help formulate potential solutions.

## Figures and Tables

**Table 1:** Potential contributing factors to LRR identified in studied patients (n **=** 10).

Potential contributing factor*	Caucasian (n = 8)	AA (n = 2)
Lack of insurance causes diagnosis delay.	4	0
Late relapses (>5 years) after original diagnosis.	3	1
Patient’s denial of breast cancer diagnosis.	4	0
Locally advanced breast cancer at presentation.	3	0

* More than one factor was noted in several patients.

## References

[1] Pierce L, Fowble B, Solin LJ, Schultz DJ, Rosser C, Goodman RL (1992). Conservative surgery and radiation therapy in black women with early stage breast cancer: patterns of failure and analysis of outcome. Cancer.

[2] Fowble BL, Schultz DJ, Overmoyer B (1994). The influence of young age on outcome in early stage breast cancer. Int. J. Radiat. Oncol. Biol. Phys..

[3] Bauer KR, Brown M, Cress RD, Parise CA, Caggiano V (2007). Descriptive analysis of estrogen receptor (ER)-negative, progesterone receptor (PR)-negative, and HER2-negative invasive breast cancer, the so-called triple-negative phenotype: a population-based study from the California cancer Registry. Cancer.

[4] Bickell NA, LePar F, Wang JJ (2007). Lost Opportunities: Physicians’ Reasons and Disparities in Breast Cancer Treatment. Journal of Clinical Oncology.

[5] Bickell NA, Wang JJ, Oluwole S (2006). Missed Opportunities: Racial Disparities in Adjuvant Breast Cancer Treatment. Journal of Clinical Oncology.

[6] Newman LA (2005). Breast Cancer in African-American Women. The Oncologist.

